# Early and Quantitative Assessment of Myocardial Deformation in Essential Hypertension Patients by Using Cardiovascular Magnetic Resonance Feature Tracking

**DOI:** 10.1038/s41598-020-60537-x

**Published:** 2020-02-27

**Authors:** Huina Liu, Jiajia Wang, Yukun Pan, Yinghui Ge, Zhiping Guo, Shihua Zhao

**Affiliations:** 1 Department of Radiology, Zhengzhou University People’s Hospital, Central China Fuwai Hospital, Heart Center of Henan Provincial People’s Hospital, Zhengzhou, Henan 450003 People’s Republic of China; 2 Department of Cardiac MR, Fuwai Hospital, National Center for Cardiovascular Diseases of China, Chinese Academy of Medical Sciences and Peking Union Medical College, No. 167 Beilishi Road, Beijing, 100037 People’s Republic of China

**Keywords:** Cardiology, Medical research

## Abstract

The aims of the study were to identify subclinical global systolic function abnormalities and evaluate influencing factors associated with left ventricular (LV) strain parameters in hypertensive subjects using cardiovascular magnetic resonance imaging feature tracking (CMR-FT). The study enrolled 57 patients with essential hypertension (mean age: 43.04 ± 10.90 years; 35 males) and 26 healthy volunteers (mean age: 38.69 ± 10.44 years; 11 males) who underwent clinical evaluation and CMR examination. Compared with controls, hypertensive patients had significantly impaired myocardial strain values while ejection fraction (EF) did not differ. After multivariate regression analyses adjustment for confounders, the global radial strains (GRS) was independently associated with the mean arterial pressure (MAP) and left ventricular mass index (LVMI) (*β* = −0.219, *p* = 0.009 and *β* = −0.224, *p* = 0.015, respectively; Adjusted *R*^2^ = 0.4); the global circumferential strains (GCS) was also independently associated with the MAP and LVMI (*β* = 0.084, *p* = 0.002 and *β* = 0.073, *p* = 0.01, respectively; Adjusted *R*^2^ = 0.439); the global longitudinal strains (GLS) was independently associated with the Age and MAP (*β* = 0.065, *p* = 0.021 and *β* = 0.077, *p* = 0.009, respectively; Adjusted *R*^2^ = 0.289). Myocardial strain can early detect the myocardial damage and may be an appropriate target for preventive strategies before abnormalities of EF.

## Introduction

Essential hypertension is defined as a rise in blood pressure which can increase risks for cerebral, cardiac, and renal events with unknown reason^1^. Hypertension remains deserving for more attention since it is a potent risk factor for cardiovascular diseases and it may brings considerable morbidity and mortality^1–4^. Given the prevalence of hypertension in the population, early identifying subclinical left ventricular (LV) systolic dysfunction among hypertensive subjects might have an important role in assessing the prognosis and choosing treatment strategies.Strain imaging has emerged as a sensitive and powerful tool to detect early and subtle myocardial dysfunction in various cardiac diseases, and may provide a novel method for LV risk assessment in hypertensive patients^5–7^.

Myocardial strain was first used by cardiovascular magnetic resonance (CMR) tagging in 1988^8^. CMR tagging has been the most available and reproducible method to quantify the myocardial deformation and remains the reference standard for evaluating myocardial strain^9,10^. However, CMR tagging needs to acquire additional sequences and its post processing is time-consuming. The spatial resolution of tagging is quite low, which is another limitation of tagging. Therefore, CMR tagging has not yet been widely applied in clinical circumstances. Speckle tracking echocardiography (STE) has solved these issues to a large extent, but it is still limited by the observer dependency, signal noise, angle dependency, inadequate acoustic windows and poor image quality^11–13^. Cardiovascular magnetic resonance imaging feature tracking (CMR-FT), which derives from cine balanced steady-state free precession (bSSFP) sequence, has the advantages of measuring myocardial strain with no need for contrast agents or additional sequence acquisition, a wide field of view, no anatomic plane restriction, semi-automatic and time-saving post-processing procedure^14–16^. Hence, CMR-FT has been increasingly used as a novel non-invasive technique for the quantitative evaluation of myocardial function^17^.

The aims of the study were to identify subclinical global systolic function abnormalities in hypertensive subjects using CMR-FT, and evaluate influencing factors associated with left ventricular strain parameters for hypertensive patients.

## Results

### Study population

The study has comprised of 57 hypertensive patients (Age: 43.04 ± 10.90 y; 35 males) and 26 healthy volunteers (Age: 38.69 ± 10.44 y; 11 males). The distributions of clinical and demographic parameters of the two groups showed in Table 1. Systolic blood pressure (SBP), diastolic blood pressure (DBP) and mean arterial pressure (MAP) of the hypertension group were significantly higher than the healthy group, and hypertensive patients tended to have higher weight, body mass index (BMI), body surface area (BSA) and dyslipidemia compared with healthy group.

**Table 1:** Distributions of clinical and demographic parameters of the study population.

	Hypertensivepatients (*n* = 57)	Healthy group (*n* = 26)	*P* value
Age (year)	43.04 ± 10.90	38.69 ± 10.44	0.092
Male (%)	35(61.4)	11 (42.3)	0.105
Height (m)	1.68 ± 0.08	1.67 ± 0.07	0.389
Weight (kg)	76.09 ± 14.28	64.81 ± 10.10	<0.001
BMI (kg/m^2^)	26.74 ± 3.37	23.33 ± 3.06	<0.001
BSA (m^2^)	1.88 ± 0.21	1.72 ± 0.16	0.001
Clinic SBP (mmHg)	146.11 ± 13.44	110.96 ± 6.00	<0.001
Clinic DBP (mmHg)	92.63 ± 10.29	71.96 ± 4.78	<0.001
MAP (mmHg)	110.46 ± 10.76	84.96 ± 4.84	<0.001
Totalserumcholesterol (mmol/l)	4.56 ± 0.81	4.05 ± 0.66	0.006
Triglycerides (mmol/l)	1.58 (1.13–2.53)	1.33 (1.01–1.64)	0.044
LDL cholesterol (mmol/l)	2.49 ± 0.75	2.14 ± 0.49	0.014
HDL cholesterol (mmol/l)	1.02 ± 0.28	1.35 ± 0.25	<0.001
Hemoglobin A1C (%)	5.74 ± 0.41	5.67 ± 0.35	0.373
Fasting blood glucose(mmol/l)	4.68 ± 0.56	4.76 ± 0.43	0.490
Heart rate (beats/min)	72.73 ± 9.90	68.35 ± 7.90	0.051

Comparison of variables between the two groups was performed by using the *Mann-Whitney U* test for triglycerides, the *Chi square* test for sex, and the independent-sample Student’s *t* test for other variables. BMI, body mass index; BSA, body surface area; SBP, systolic blood pressure; DBP, diastolic blood pressure; MAP, mean arterial pressure, LDL cholesterol, low density lipoprotein cholesterol; HDL cholesterol, high density lipoprotein cholesterol.

### Myocardial deformation by feature tracking

There were no significant differences in LV end-diastolic volume indexed (EDVI), LV end systolic volume indexed (ESVI) and LVEF between the two groups, except for LV mass indexed (LVMI) (Table 2).

**Table 2:** Comparison of left ventricular parameters of CMR in two groups.

	Hypertensivepatients (*n* = 57)	Healthy group (*n* = 26)	*P* value
Indexed LVEDV (ml/m^2^)	63.59 ± 10.17	66.71 ± 7.71	0.168
Indexed LVESV (ml/m^2^)	23.54 ± 5.88	23.09 ± 3.86	0.678
LVEF (%)	63.19 ± 6.18	65.42 ± 4.13	0.056
Left ventricle mass/Height (g/m)	70.49 ± 13.24	53.38 ± 9.21	<0.001
Left ventricle mass /BSA (g/m^2^)	63.13 ± 10.79	51.47 ± 7.05	<0.001
GRS (%)	35.14 ± 7.27	42.14 ± 7.80	<0.001
GCS (%)	−19.64 ± 2.46	−21.87 ± 2.42	<0.001
GLS (%)	−16.48 ± 2.34	−17.85 ± 1.83	0.010

Comparison of variables between the two groups was performed by using the independent-sample Student’s t test. Indexed LVEDV, Indexed left ventricular enddiastolic volume; Indexed LVESV, Indexed left ventricular end-systolic volume; LVEF, left ventricular ejection fraction; BSA, body surface area; GRS, global radial strain; GCS, global circumferential strain; GLS, global longitudinal strain.

**Figure 1:**
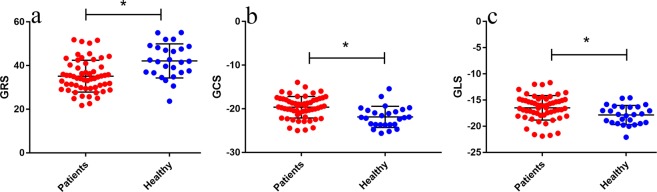
Scatter dot plots reporting the comparison of the differences in hypertensive patients and healthy controls for global radial strain (GRS) (**a**), global circumferential strain (GCS) (**b**) and global longitudinal strain (GLS) (**c**). The black lines represents the mean with *SD*. The GRS, GCS and GLS were significantly different between the two groups. *P < 0.05. GRS indicates global radial strain; GCS indicates global circumferential strain; GLS indicates global longitudinal strain.

**Figure 2:**
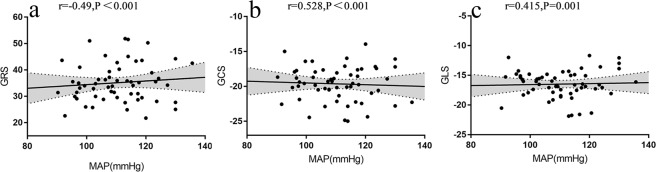
Scatter plots of global radial strain (GRS) (**a**), global circumferential strain (GCS) (**b**) and global longitudinal strain (GLS) (**c**) against mean arterial pressure (MAP), linear regression estimates with 95% confidence limits (black lines with gray shades). GRS indicates global radial strain; GCS indicates global circumferential strain; GLS indicates global longitudinal strain; MAP indicates mean arterial pressure.

Compared with the control group, hypertensive patients had significantly reduced LV myocardial deformations mirrored by global radial strains (GRS), global circumferential strains (GCS) and global longitudinal strains (GLS) (Table 2, Fig. 1). In univariable linear regression analyses in the hypertensive population, reduced GRS, GCS and GLS were all associated with MAP (*β* = −0.331, 0.121, and 0.09, respectively) and LVMI (LVM/BSA) (*β* = −0.366, 0.125, and 0.081, respectively) (Table 3, Fig. 2).

**Table 3:** Univariable correlations of GRS, GCS and GLS in the hypertensive patients.

	GRS		GCS		GLS	
	*β*	*P* value	*β*	*P* value	*β*	*P* value
Age (year)	−0.037	0.681	0.014	0.656	0.045	0.12
Male (%)	−4.288	0.029	1.336	0.045	1.396	0.027
Height (m)	−33.115	0.006	10.727	0.009	9.355	0.017
Weight (kg)	−0.146	0.031	0.047	0.039	0.041	0.059
BMI (kg/m^2^)	−0.372	0.201	0.119	0.224	0.107	0.255
BSA (m^2^)	−10.446	0.02	3.373	0.026	2.968	0.04
Clinic SBP (mmHg)	−0.228	0.001	0.086	<0.001	0.075	0.001
Clinic DBP (mmHg)	−0.348	<0.001	0.124	<0.001	0.084	0.005
MAP (mmHg)	−0.331	<0.001	0.121	<0.001	0.090	0.001
Totalserumcholesterol (mmol/l)	−1.949	0.103	0.535	0.188	0.299	0.441
LDLcholesterol (mmol/l)	−0.917	0.487	0.285	0.523	0.123	0.773
HDL cholesterol (mmol/l)	6.334	0.07	−2.416	0.04	−0.815	0.474
Hemoglobin A1C (%)	−1.374	0.563	0.638	0.426	0.257	0.737
Fasting blood glucose (mmol/l)	−1.188	0.497	0.323	0.585	0.584	0.298
Heart rate (beats/min)	−0.148	0.133	0.059	0.077	0.023	0.466
Left ventricle mass/Height (g/m)	−0.314	<0.001	0.107	<0.001	0.072	0.002
Left ventricle mass /BSA (g/m^2^)	−0.366	<0.001	0.125	<0.001	0.081	0.004

GRS, global radial strain; GCS, global circumferential strain; GLS, global longitudinal strain; BMI, body mass index; BSA, body surface area; SBP, systolic blood pressure; DBP, diastolic blood pressure; MAP, mean arterial pressure, LDL cholesterol, low density lipoprotein cholesterol; HDL cholesterol, high density lipoprotein cholesterol.

After multivariable regression analyses adjustment for confounders including age, sex, BSA, high density lipoprotein cholesterol (HDLC) and LVMI, the associations with MAP remained significant for GRS, GCS and GLS (Table 4). In multivariable regression models, GRS and GCS had a negative association with MAP and LVMI; a negative association was revealed between GLS and Age, but the association of GLS with LVMI was attenuated after adjustment for confounders, however, GLS maintained a negative association with MAP (Table 4). The above results showed that the increased blood pressure may have an adverse effect on myocardial strains, and the decrease of GRS and GCS strains was also associated with the increased LVMI, and GLS may decrease with age.

**Table 4:** Multiple Linear Regression Analysis of Left Ventricular Strain in Hypertension Group.

	GRS		GCS		GLS	
	(*R*^2^ = 0.464, Adjusted *R*^2^ = 0.4, *F* = 7.219, *P* < 0.001)		(*R*^2^ = 0.499, Adjusted *R*^2^ = 0.439 *F* = 8.291, *P* < 0.001)		(*R*^2^ = 0.353, Adjusted *R*^2^ = 0.289 *F* = 5.563, *P* < 0.001)	
	*β*	*P* value	*β*	*P* value	*β*	*P* value
Age (year)	−0.054	0.496	0.018	0.474	0.065	0.021
Male	0.075	0.972	−0.12	0.866	0.579	0.446
BSA (m^2^)	−9.132	0.071	3.013	0.068	3.198	0.07
MAP (mmHg)	−0.219	0.009	0.084	0.002	0.077	0.009
HDL cholesterol (mmol/l)	4.254	0.129	−1.744	0.058	—	—
Left ventricle mass/BSA(g/m^2^)	−0.224	0.015	0.073	0.015	0.016	0.602

GRS, global radial strain; GCS, global circumferential strain; GLS, global longitudinal strain; BSA, body surface area; MAP, mean arterial pressure, HDL cholesterol, high density lipoprotein cholesterol.

**Figure 3:**
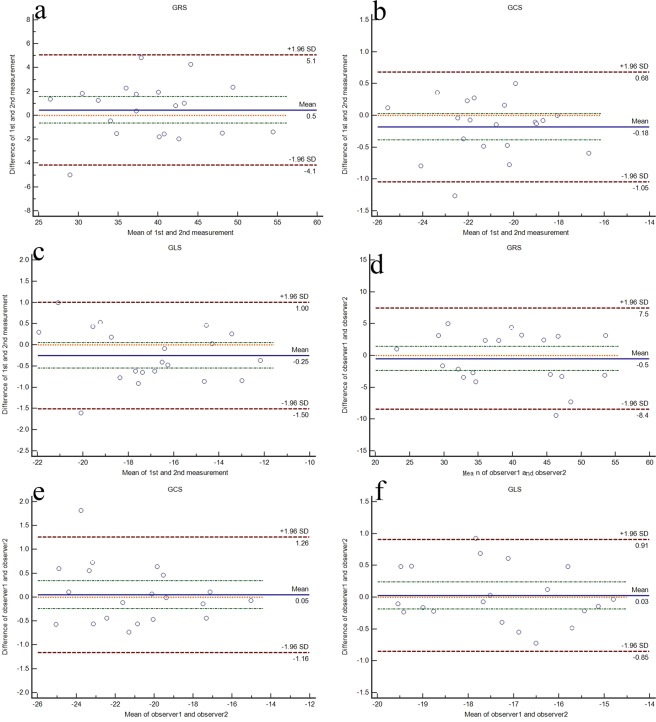
*Bland-Altman* plots of the intra- and inter-observer reproducibility for GRS (**a,d**), GCS (**b,e**) and GLS (**c,f**) measured by CMR-FT. The *Bland-Altman* plots include the line of equality, the line of mean difference, the lines of the 95% confidence interval of mean of differences and the lines of the 95% limits of agreement. GRS indicates global radial strain; GCS indicates global circumferential strain; GLS indicates global longitudinal strain.

In our study, all segments were available to be tracked. Intra- and inter-observer variabilities were showed in Table 5. All strain parameters had intra- and inter-observer *ICC* of ≥0.85, the reproducibility of the circumferential and longitudinal strain was better than that of the radial strain. The *Bland-Altman* plots of intra- and inter-observer agreement were displayed in Fig. 3.

**Table 5:** Intra- and inter-observer agreement in strain measurement.

	Mean difference	Limits of agreement	*ICC*(95% *CI*)
**GRS**			
Intra-observer	0.46	−4.15–5.07	0.946 (0.871–0.978)
Inter-observer	−0.49	−8.44–7.46	0.898 (0.762–0.958)
**GCS**			
Intra-observer	−0.18	−1.05–0.68	0.978 (0.944–0.991)
Inter-observer	0.05	−1.16–1.26	0.977 (0.939–0.991)
**GLS**			
Intra-observer	−0.25	−1.50–1.00	0.970 (0.923–0.988)
Inter-observer	0.03	−0.85–0.91	0.960 (0.902–0.984)

GRS, global radial strain; GCS, global circumferential strain; GLS, global longitudinal strain.

## Discussion

The main findings of the study were that (1) The hypertensive patients were inclined to have higher weight, BMI, BSA, serum lipids and LV mass. (2) The myocardial strain valueshad already significantly impaired before EF, EDVI and ESVI appeared abnormal in hypertensive patients. (3) There were a negative association between MAP and reduction of GRS, GCS and GLSwhen adjusting for key confounders of LV myocardial dysfunction, including age, sex, BSA, HDLC and LVMI in hypertensive patients. (4) All the strain parameters above had good reproducibility.

LVEF has been recognized as the routine prognostic parameter and reference standard of LV global systolic function. However, LVEF is only a representative of LV geometric change other than the function change^18^, which indicates EF may be inaccessible to identify subtle and mild degrees of LV systolic abnormalities^19–23^. Maciver *et al*. reported that although longitudinal systolic strain had been reduced, EF may be normal by compensated with the increased radial wall thickness in LV hypertrophy^24^. Our results showed that hypertensive patients had significantly reduced myocardial strains in early normal EF stage compared with control subjects, which confirmed the value of the strain in detection of early and subtle myocardial impairment with previous studies^25–29^.

The reason that reduced GRS, GCS and GLS were all independently associated with MAP may be that long-standing hypertension cause microscopic changes such as increased collagen turnover, myocardial fibrosis and subendocardial ischemia which lead to impaired strain, as other study explained^30,31^. Saeed *et al*.^27^ reported reduced GLS was particularly associated with hypertension and Navarini *et al*.^25^ reported 3D strains had the similar results. Our results indicated GRS and GCS both had a negative association with LVMI (LVM/BSA). Higher BP could result in LV hypertrophy (LVH) which had increased LVMI^32^. LV hypertrophy secondary to pressure overload is a compensation for high intracavitary pressures for maintaining normal wall stress and cardiac output^33–35^. According to our results, the process of myocardial hypertrophy may have been accompanied by the decrease of radial and circumferential strains. In addition, our results showed that GLS may decrease with age. Kaku *et al*. also found that GLS have significant age dependency, with the highest value in infancy and a gradual reduction with age^36^. The age-related association with GLS may reveal the process of myocardial maturation and aging. Recently, the association of reduced strain with poor prognosis in hypertension was reported by Lee *et al*.^37^. Despite the established role of LVEF and increased LV mass for higher incidence of cardiovascular risk^20,32,35,38^, more and more studies showed strain (especially LV longitudinal strain) was superior and incremental for assessing the relation with major adverse cardiac events (MACE)^39–41^.

The normal reference values of strain parameters by using CMR-FT varied in different study. The discrepancies among different studies may attribute to different applied vendor or software version, as well as different patient characteristics such as age, sex, BMI, BP and HR^42–44^. For example, for healthy adults, Hwang *et al*. reported left ventricular GCS value was comparable to our result, but the values of left ventricular GRS and GLS were higher than that in our study^45^. Thus, the normalization and standardization of strain parameters are required. In recent years, the reproducibility of LV strains by using CMR-FT had been proved^46–48^. Our results showed good reproducibility for all global strain parameters with intra- and inter-observer ICC of ≥0.85, the reproducibility of the circumferential and longitudinal strains was better than that of the radial strain. Our outcomes were consistent with the previous studies^16,46,49,50^. The good reproducibility of the strain may owe to the advantages of superior image quality and accurate definition of the endocardial border, as well as the algorithm by using automated process and background smoothing^17,46^. For the comparatively lower reproducibility of radial strain, the likely reason is that radial strain is derived from both endocardial and epicardial motion which is different with circumferential strain and longitudinal strain, and that the contrast of the epicardial signal intensity is less clear than that of the endocardial signal intensity^46^. In addition, the accuracy of the strain parameters by using CMR-FT could be validated by comparing with CMR tagging. Good agreement with CMR tagging had been proved^14,51^.

## Limitations

The main limitations of this study were that: First, our study was limited by the relatively small number of subjects, which made it impossible for subgroup analysis with and without LV hypertrophy. Second, although several factors were incorporated toevaluate the associations with strains, there are other possible important confounders such as the application of antihypertensive drugs that have not yet been assessed. Thus, our results should be interpreted in the circumstances of the limitations, and it may be difficult to generalize our results. Of course, larger patient populations and more confounders should be performed to confirm our initial results.

## Conclusions

Myocardial strain can early detect the myocardial damage and may be an appropriate target for preventive strategies because it occurs before abnormalities of EF. Among essential hypertensive patients, increased MAP may have an adverse effect on LV myocardial strains. CMR-FT may have potential to quantitatively analyze cardiac motion and apply in routine clinical practice in the future.

## Methods

### Study population

The prospective study enrolled 57 participants with a diagnosis of essential hypertension from our hospital between October 2017 and October 2018. The patients had normal EF (≥50%) and no history or clinical evidence of cardiovascular disease. Hypertension was defined as a history of hypertension, use of antihypertensive medicines, or the values ≥130 mmHg SBP and/or ≥80 mmHg DBP according to the 2017 guideline^52^. The patients had BP measurements using an automated electronic sphygmomanometer (HBP-9020, OMRON, Dalian, China). According to the 2017 guideline^52^, after a minimum 5-min rest without caffeine, exercise, or smoking for at least 30 min in sitting position, blood pressure measurements were performed three times consecutively at 1–2 min intervals. Then, the individual’s level of BP was calculated by the average of three measurements. Clinical and laboratory data were also obtained. The exclusion criteria includedsymptoms of chest pain or a history of coronary heart disease, myocardial infarction, cardiomyopathy, severe valvular disease, arhythmia, heart failure, severe obstructive lung disease, stroke, diabetes, kidney disease, thyroid disease and hypohemia. The patients who had contraindications for CMR such as claustrophobia, implantable not CMR safe materials, severe mental disorder were also excluded. In addition, we recruited 26 healthy volunteers as a control group for matching age and sex with case group. Volunteers with any of the symptoms and signs or a history of cardiovascular diseases, cerebrovascular diseases or relevant noncardiac diseases were excluded. All of the volunteers had normal blood test results.Written informed consent was obtained from all study participants. The study was approved by the ethics committee of central China Fuwai hospital. All methods were performed in accordance with the guidelines and regulations of the Declaration of Helsinki.

### CMR image acquisition

All imaging was performed on a 3.0 Tesla MRI scanner (Magnetom Skyra, Siemens Healthcare, Erlangen, Germany) using an 18-channel cardiac phased-array receiver coil with patients in the supine position. Cine images were acquired using a standard balanced steady state free precession (bSSFP) sequence with retrospective electrocardiogram-gating in short axis (SAX) views from base to apex of the LV and long axis 2-, 3- and 4-chamber views. To cover the entire LV, 8 to 12 short-axis slices were performed with 4 to 6 breath-holds. All cine images were acquired with 25 phases per cardiac cycle. The scan parameters were: repetition time (TR) 3.2 ms, echo time (TE)1.40 ms, temporal resolution 44.66 ms, spatial resolution 1.8 × 1.8 mm, field of View (FOV) 369 × 369 mm, matrix 208 × 146, flip angle 49°, slice thickness 8 mm, slice gap 2 mm, acceleration factor 3.

### CMR strain analysis

#### Feature tracking

**Figure 4:**
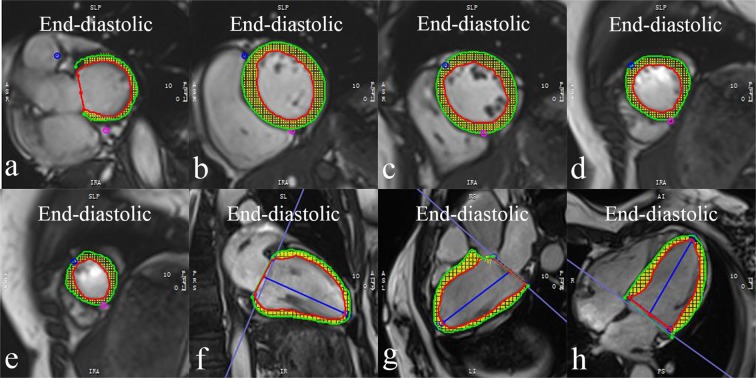
LV feature tracking analysis (1) Iidentify end-diastolic phase (reference phase); (2) The endo- and epicardial borders were automatically drew in the end-diastolic phase using SAX slices (**a-e**) and long axis 2-, 3- and 4-chamber slices (**f**–**h**), which could be manually adjusted if poor tracking; (3) Then, the software algorithm automatically drew the borders and traced the myocardium voxel points throughout the other cardiac phases during a cardiac cycle based on the endo- and epicardial contours of reference phase; (4) Finally, the 2D strain values were calculated automatically.

Images were analysed by an experienced radiologist blinded to the patients’ situations. CMR-FT analysis was performed using dedicated post-processing software (CVI^42^ version 5.9.1, Circle Cardiovascular Imaging, Calgary, Canada). The endo- and epicardial border lines were marked automatically and trimmed manually in the end-diastolic phase (reference phase) on SAX slices. By using a maximum likelihood method, the software algorithm automatically drew the borders and traced the myocardium voxel points throughout the other cardiac phases during a cardiac cycle based on the endo- and epicardial contours of reference phase. The borders could be manually adjusted if inadequate tracking. Finally, the 2D strain values were calculated with the movement of the features relative to the reference phase^53^ (Fig. 4). The short axis cine images were used to obtain GCS and GRS. The 4-chamber, 2-chamber, 3-chamber long-axis cine images were used to obtain GLS. LV volumes (end-diastolic and end-systolic volumes), LV myocardial mass and function (LVEF) were calculated from the short-axis cine images.

#### Reproducibility

Intra- and inter-observer variabilities of the measurements of GRS, GCS and GLS of 20 subjects randomly selected from the two groups (10 patients and 10 volunteers) were evaluated by two independent blinded observers. For intra-observer variability, the interval time between the two measurements is one month. For inter-observer variability, the two separate observers were blind to each other’s measurements.

#### Statistical analyses

Statistical analyses were performed with SPSS statistics 22 (IBM corporation, USA) and MedCalc15.2.2 (MedCalc Software, Ostend, Belgium). Normal distribution of variables was assessed by *Shapiro-Wilk W* test. Continuous variables with normal distribution were presented as mean ± standard deviations, continuous variables with nonnormal distribution were presented as median with interquartile range (IQR). Categorical variables were presented as percentages. Comparisons of variables between the hypertensive patients and the controls were performed by using the independent-sample Student’s *t*-test for normally distributed continuous variables, the *Mann-Whitney U* test for non-normally distributed continuous variables and the *Chi square* test for categorical variables. Univariable and multivariable linear regression analyses were used to evaluate the associations between LV strain parameters and the influencing factors. Covariates for multivariate models were selected based on the significant variables in the univariable analysis (*p* < 0.20) and clinically relevant parameters. For reproducibility evaluation, intraclass correlation coefficient (*ICC*) and *Bland-Altman* plots were calculated. A *P* value of <0.05 was considered statistically significant.

## Data Availability

The datasets used and/or analyzed during the current study available from the corresponding author on reasonable request.

## References

[1] Messerli FH, Williams B, Ritz E (2007). Essential Hypertension. Lancet..

[2] Levy D, Larson MG, Vasan RS, Kannel WB, Ho KK (1996). The Progression From Hypertension to Congestive Heart Failure. JAMA..

[3] Haider AW, Larson MG, Franklin SS, Levy D, Framingham HS (2003). Systolic Blood Pressure, Diastolic Blood Pressure, and Pulse Pressure as Predictors of Risk for Congestive Heart Failure in the Framingham Heart Study. Ann. Intern. Med..

[4] Drazner MH (2011). The Progression of Hypertensive Heart Disease. Circulation..

[5] Marwick TH (2006). Measurement of Strain and Strain Rate by Echocardiography. J. Am. Coll. Cardiol..

[6] Yang H (2003). Use of Strain Imaging in Detecting Segmental Dysfunction in Patients with Hypertrophic Cardiomyopathy. J. Am. Soc. Echocardiog..

[7] Mottram PM (2004). Effect of Aldosterone Antagonism On Myocardial Dysfunction in Hypertensive Patients with Diastolic Heart Failure. Circulation..

[8] Zerhouni EA, Parish DM, Rogers WJ, Yang A, Shapiro EP (1988). Human Heart: Tagging with MR Imaging–A Method for Noninvasive Assessment of Myocardial Motion. Radiology..

[9] Gotte MJ (2006). Myocardial Strain and Torsion Quantified by Cardiovascular Magnetic Resonance Tissue Tagging: Studies in Normal and Impaired Left Ventricular Function. J. Am. Coll. Cardiol..

[10] Shehata ML, Cheng S, Osman NF, Bluemke DA, Lima JA (2009). Myocardial Tissue Tagging with Cardiovascular Magnetic Resonance. J. Cardiovasc. Magn. Reson..

[11] Amundsen BH (2006). Noninvasive Myocardial Strain Measurement by Speckle Tracking Echocardiography. J. Am. Coll. Cardiol..

[12] Sutherland GR, Di Salvo G, Claus P, D’Hooge J, Bijnens B (2004). Strain and Strain Rate Imaging: A New Clinical Approach to Quantifying Regional Myocardial Function. J. Am. Soc. Echocardiog.

[13] Marwick TH (2009). Myocardial Strain Measurement with 2- Dimensional Speckle-Tracking Echocardiography: Definition of Normal Range. JACC Cardiovasc. Imaging..

[14] Hor KN (2010). Comparison of Magnetic Resonance Feature Tracking for Strain Calculation with Harmonic Phase Imaging Analysis. JACC Cardiovasc. Imaging..

[15] Truong UT (2010). Significance of Mechanical Alterations in Single Ventricle Patients On Twisting and Circumferential Strain as Determined by Analysis of Strain From Gradient Cine Magnetic Resonance Imaging Sequences. Am. J. Cardiol..

[16] Pedrizzetti, G., Claus, P., Kilner, P. J. & Nagel, E. Principles of Cardiovascular Magnetic Resonance Feature Tracking and Echocardiographic Speckle Tracking for Informed Clinical Use. *J. Cardiovasc. Magn. R*. **18** (2016). 10.1186/s12968-016-0269-7 PMC5000424 27561421

[17] Claus P, Omar A, Pedrizzetti G, Sengupta PP, Nagel E (2015). Tissue Tracking Technology for Assessing Cardiac Mechanics: Principles, Normal Values, and Clinical Applications. JACC Cardiovasc. Imaging.

[18] Hurlburt HM (2007). Direct Ultrasound Measurement of Longitudinal, Circumferential, and Radial Strain Using 2-Dimensional Strain Imaging in Normal Adults. Echocardiography..

[19] Marwick TH (2013). Methods Used for the Assessment of LV Systolic Function: Common Currency Or Tower of Babel?. Heart..

[20] Curtis JP (2003). The Association of Left Ventricular Ejection Fraction, Mortality, and Cause of Death in Stable Outpatients with Heart Failure. J. Am. Coll. Cardiol..

[21] Schuster A, Hor KN, Kowallick JT, Beerbaum P, Kutty S (2016). Cardiovascular Magnetic Resonance Myocardial Feature Tracking: Concepts and Clinical Applications. Circ. Cardiovasc. Imaging..

[22] Sardana M (2019). Usefulness of Left Ventricular Strain by Cardiac Magnetic Resonance Feature-Tracking to Predict Cardiovascular Events in Patients with and without Heart Failure. Am. J. Cardiol..

[23] Shah AM (2015). Prognostic Importance of Changes in Cardiac Structure and Function in Heart Failure with Preserved Ejection Fraction and the Impact of Spironolactone. Circ. Heart Fail..

[24] Maciver DH, Townsend M (2008). A Novel Mechanism of Heart Failure with Normal Ejection Fraction. Heart..

[25] Navarini S (2017). Myocardial Deformation Measured by 3-Dimensional Speckle Tracking in Children and Adolescents with Systemic Arterial HypertensionNovelty and Significance. Hypertension..

[26] Tadic M (2014). The Impact of High-Normal Blood Pressure On Left Ventricular Mechanics: A Three-Dimensional and Speckle Tracking Echocardiography Study. Int. J. Cardiovasc. Imaging..

[27] Saeed, S. *et al* Left Ventricular Myocardial Dysfunction in Young and Middle-Aged Ischemic Stroke Patients. *J. Hypertens*. **1** (2018). 10.1097/HJH.0000000000001925 30188424

[28] Haeck ML (2012). Prognostic Value of Right Ventricular Longitudinal Peak Systolic Strain in Patients with Pulmonary Hypertension. Circ. Cardiovasc. Imaging..

[29] Fine NM (2013). Outcome Prediction by Quantitative Right Ventricular Function Assessment in 575 Subjects Evaluated for Pulmonary Hypertension. Circ. Cardiovasc. Imaging..

[30] Poulsen SH, Andersen NH, Heickendorff L, Mogensen CE (2005). Relation Between Plasma Amino-Terminal Propeptide of Procollagen Type III and Left Ventricular Longitudinal Strain in Essential Hypertension. Heart..

[31] Lip GY, Felmeden DC, Li-Saw-Hee FL, Beevers DG (2000). Hypertensive Heart Disease. A Complex Syndrome Or a Hypertensive ‘Cardiomyopathy’?. Eur. Heart J..

[32] Koren MJ, Devereux RB, Casale PN, Savage DD, Laragh JH (1991). Relation of Left Ventricular Mass and Geometry to Morbidity and Mortality in Uncomplicated Essential Hypertension. Ann. Intern. Med..

[33] Hwang JW (2017). Assessment of Reverse Remodeling Predicted by Myocardial Deformation On Tissue Tracking in Patients with Severe Aortic Stenosis: A Cardiovascular Magnetic Resonance Imaging Study. J. Cardiovasc. Magn. Reson..

[34] Carabello BA, Paulus WJ (2009). Aortic Stenosis. Lancet..

[35] Levy D, Garrison RJ, Savage DD, Kannel WB, Castelli WP (1990). Prognostic Implications of Echocardiographically Determined Left Ventricular Mass in the Framingham Heart Study. N. Engl. J. Med..

[36] Kaku K (2014). Age-Related Normal Range of Left Ventricular Strain and Torsion Using Three-Dimensional Speckle-Tracking Echocardiography. J. Am. Soc. Echocardiogr..

[37] Lee W, Liu Y, Yang L, Tsai W (2016). Prognostic Value of Longitudinal Strain of Subepicardial Myocardium in Patients with Hypertension. J. Hypertens..

[38] Devereux RB (2004). Prognostic Significance of Left Ventricular Mass Change During Treatment of Hypertension. JAMA..

[39] Saito M (2016). Prognostic Implications of LV Strain Risk Score in Asymptomatic Patients with Hypertensive Heart Disease. JACC Cardiovasc. Imaging..

[40] Kalam K, Otahal P, Marwick TH (2014). Prognostic Implications of Global LV Dysfunction: A Systematic Review and Meta-Analysis of Global Longitudinal Strain and Ejection Fraction. Heart..

[41] Arenja N (2017). Diagnostic and Prognostic Value of Long-Axis Strain and Myocardial Contraction Fraction Using Standard Cardiovascular MR Imaging in Patients with Nonischemic Dilated Cardiomyopathies. Radiology..

[42] Dalen H (2010). Segmental and Global Longitudinal Strain and Strain Rate Based On Echocardiography of 1266 Healthy Individuals: The HUNT Study in Norway. Eur. J. Echocardiogr..

[43] Yingchoncharoen T, Agarwal S, Popovic ZB, Marwick TH (2013). Normal Ranges of Left Ventricular Strain: A Meta-Analysis. J. Am. Soc. Echocardiogr..

[44] Sugimoto T (2017). Echocardiographic Reference Ranges for Normal Left Ventricular 2D Strain: Results From the EACVI NORRE Study. Eur. Heart J. Cardiovasc. Imaging..

[45] Hwang JW, Cha MJ, Kim SM, Kim Y, Choe YH (2018). Relationship Between Cardiovascular Risk Factors and Myocardial Strain Values of Both Ventricles in Asymptomatic Asian Subjects: Measurement Using Cardiovascular Magnetic Resonance Tissue Tracking. Int. J. Cardiovasc. Imaging..

[46] Taylor RJ (2015). Myocardial Strain Measurement with Feature-Tracking Cardiovascular Magnetic Resonance: Normal Values. Eur. Heart J. Cardiovasc. Imaging..

[47] Schmidt B (2017). Intra- and Inter-Observer Reproducibility of Global and Regional Magnetic Resonance Feature Tracking Derived Strain Parameters of the Left and Right Ventricle. Eur. J. Radiol..

[48] Maceira AM (2018). Feasibility and Reproducibility of Feature-Tracking-Based Strain and Strain Rate Measures of the Left Ventricle in Different Diseases and Genders. J. Magn. Reson. Imaging..

[49] Zitzelsberger, T. *et al* Magnetic Resonance-Based Assessment of Myocardial 2-Dimensional Strain Using Feature Tracking: Association with Cardiovascular Risk Factors in a Population-Based Cohort Free of Cardiovascular Disease. *J. Thorac. Imaging*. (2018). 10.1097/RTI.0000000000000380 30570523

[50] Hu, L. *et al* Assessment of Global and Regional Strain Left Ventricular in Patients with Preserved Ejection Fraction After Fontan Operation Using a Tissue Tracking Technique. *Int. J. Cardiovasc. Imaging.* (2018). 10.1007/s10554-018-1440-z 30121757

[51] Moody WE (2015). Comparison of Magnetic Resonance Feature Tracking for Systolic and Diastolic Strain and Strain Rate Calculation with Spatial Modulation of Magnetization Imaging Analysis. J. Magn. Reson. Imaging..

[52] Whelton PK (2018). ACC/AHA/AAPA/ABC/ACPM/AGS/APhA/ASH/ASPC/NMA/PCNA Guideline for the Prevention, Detection, Evaluation, and Management of High Blood Pressure in Adults: A Report of the American College of Cardiology/American Heart Association Task Force on Clinical Practice Guidelines. Circulation..

[53] Andre F (2015). Age- and Gender-Related Normal Left Ventricular Deformation Assessed by Cardiovascular Magnetic Resonance Feature Tracking. J. Cardiovasc. Magn. Reson..

